# Quadricuspid aortic valve and a ventricular septal defect in a horse

**DOI:** 10.1186/1746-6148-10-142

**Published:** 2014-06-30

**Authors:** Katarzyna M Michlik, Anna K Biazik, Radomir Z Henklewski, Marta A Szmigielska, Józef M Nicpoń, Urszula Pasławska

**Affiliations:** 1 Department of Internal Medicine and Clinic of Disease of Horses, Dogs and Cats, Veterinary Faculty of Wrocław University of Environmental and Life Sciences, pl. Grunwaldzki 47, 50-366 Wrocław, Poland; 2 Department and Clinic of Surgery, Veterinary Faculty of Wrocław University of Environmental and Life Sciences, pl. Grunwaldzki 51, 50-366 Wrocław, Poland

**Keywords:** Horse, Quadricuspid aortic valve, Ventricular septal defect, Congenital heart defect, Echocardiography

## Abstract

**Background:**

Quadricuspid aortic valve (QAV) and ventricular septal defect (VSD) are congenital heart defects and have been described in both human and veterinary medical literature.

**Case presentation:**

A 5-year-old half-bred bay stallion was referred for surgical castration. Cardiac murmurs were heard on the presurgical clinical examination and the cardiac examination revealed subcutaneous oedema, tachycardia with a precodrial thrill and a grade 5/6 pansystolic murmur, which was heard on auscultation of the right and left side of the chest. Examination of the B-mode echocardiograms revealed the presence of a QAV (one small cusp, two equal-sized cusps, and one large cusp) and VSD in the membranous portion of the intraventricular septum. These two congenital cardiac defects were accompanied by mild aortic valve regurgitation and severe tricuspid regurgitation. Despite the presence of these cardiac defects, the horse underwent surgical castration under general anesthesia. Surgery, anaesthesia and recovery from anaesthesia were uneventful. The gelding was euthanasied after 17 months because of a progressive loss of body weight, weakness and recumbency.

**Conclusion:**

A QAV in combination with VSD in a horse is an interesting finding, because to the best of our knowledge, this has not been previously described in equine literature.

## Background

A quadricuspid aortic valve (QAV) is a congenital heart defect in which four morphologically anomalous cusps of the aortic valve occur. In human medical literature, QAVs have been classified on the basis of a differentiated morphology of the cusps [1]. QAV is usually diagnosed as a single cardiac anomaly, but can also be associated with other cardiac defects. In veterinary medicine, QAV with concomitant VSD has been described in dogs [2-4], in a shrew [5] and in hamsters [6]. A QAV is usually diagnosed with echocardiography. Physical examination usually reveals a diastolic heart murmur and no symptoms of heart disease [7].

Ventricular septal defects (VSDs) have been described both in humans and animals. VSDs are classified according to their location [8]. It is the most commonly diagnosed congenital heart disease in horses, among which Arabian horses exhibit particular predisposition to this disorder [9]. It may occur as a single defect, but it is often a part of a complex congenital anomaly [10-14], among which the best known congenital syndrome is the tetralogy of Fallot [11,15-17].

The reference non-invasive method to diagnose both VSD and QAV is echocardiography. The purpose of the present case report is to describe the clinical and echocardiographic findings in a horse presenting concurrent QAV and VSD.

## Case presentation

**Figure 1:**
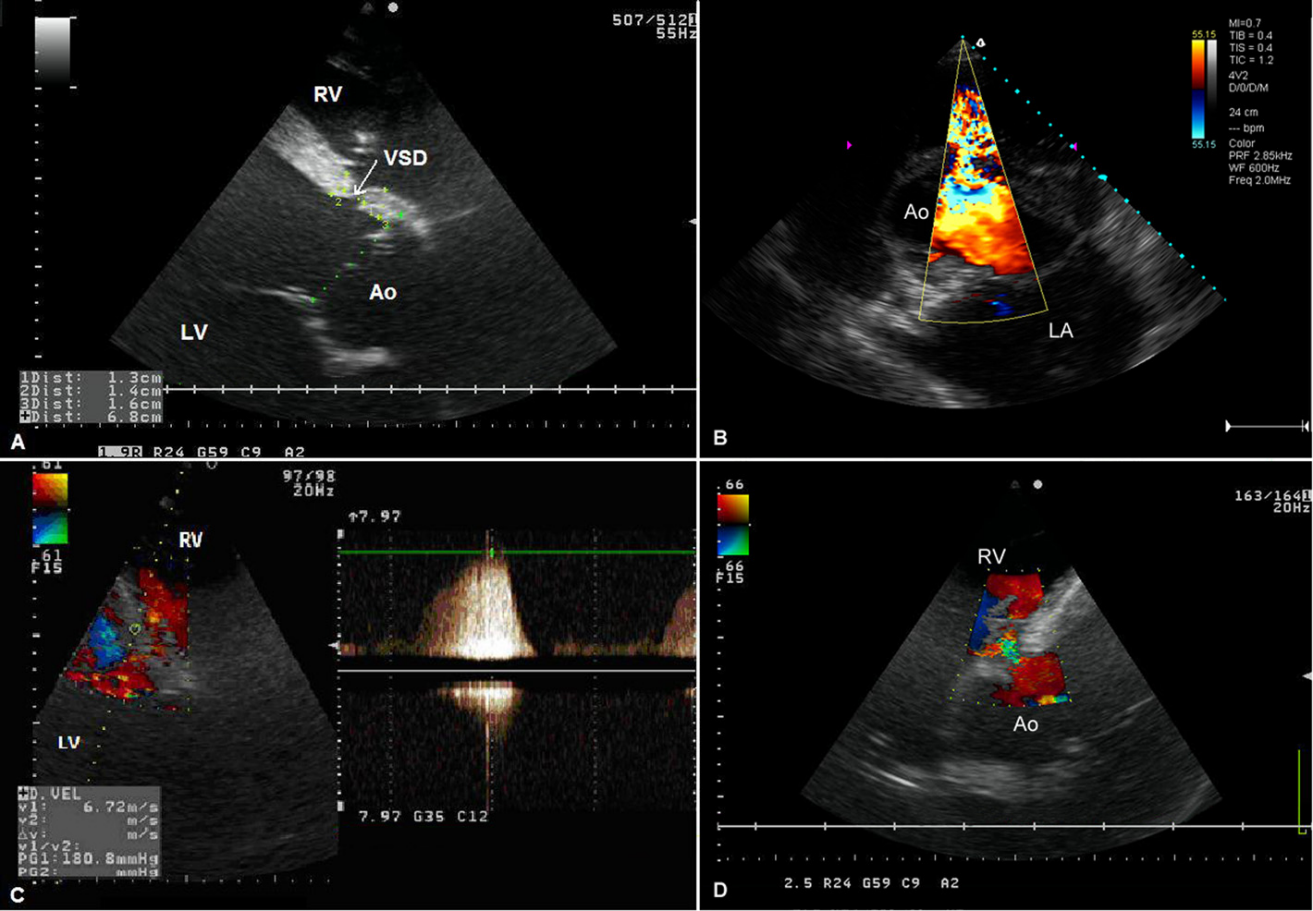
**Ventricular septal defect. A:** A long-axis image of the left ventricular outflow tract which was obtained by transthoracic echocardiography when the transducer was placed in the fourth right intercostal space. Ao – aorta, VSD – ventricular septal defect, RV – right ventricle, LV – left ventricle. **B:** A transverse view at the level of the aorta and left atrium, which was obtained by transthoracic color Doppler echocardiography when the transducer was placed in the third right intercostal space. The ventricular septal defect in which blood flows from the left ventricle into the right ventricle is seen as a multicolored shunt. Ao – aorta, LA – left atrium. **C:** A multicolored long axis image of the shunt jet caused by the turbulent blood flow through VSD which was obtained by continuous-wave Doppler echocardiography when the transducer was placed in the fourth right intercostal space. The maximum velocity through the VSD was 6.72 m/sec. RV – right ventricle, LV – left ventricle. **D:** A long-axis image of the left ventricular outflow tract which was obtained by transthoracic color Doppler echocardiography when the transducer was placed in the fourth right intercostal space. Ao – aorta, RV – right ventricle.

A 5-year-old half-bred bay stallion, weighing approximately 430 kg and with general emaciation, was referred to the Department of Surgery at the University of Environmental and Life Sciences, Wroclaw, Poland for surgical castration. Cardiac murmurs were heard on clinical examination prior to the surgery, and a cardiologic consultation was subsequently performed. The physical examination revealed (a) loss of muscle mass, which the owner reported as being progressive, (b) hypertrophic pododermatitis (equine canker) in all four limbs, (c) subcutaneous oedema and (d) a precordial thrill and a grade 5/6 pansystolic murmur, which was best heard on auscultation in the fourth right intercostal space. A holosystolic heart murmur with its point of maximum intensity in the third left intercostal space was also heard on auscultation. A standard electrocardiographic examination using an analog electrocardiograph (Schiller AT-1, Schiller AG, Switzerland, Europe) revealed a sinus tachycardia of 56 beats/minute without any arrhythmia. An echocardiographic examination was then carried out according to a previously published protocol [18,19] using an ultrasound system (Terason t3000™, Burlington, MA, USA) with a 2.5 MHz phased-array transducer with harmonic imaging. The B-mode right parasternal long axis left ventricular outflow views revealed the presence of a VSD in the membranous portion of the intraventricular septum (Figure 1A). Its presence was confirmed by transthoracic color Doppler echocardiography in the right parasternal long axis view (Figure 1D) and transverse view at the level of the aorta and left atrium (Figure 1B). In both the long-axis and short-axis, images of the left ventricular outflow tract, which were obtained when the transducer was placed in the fourth right intercostal space, the largest measured diameter of the VSD was 1.6 cm and the end-diastolic diameter of the aorta was 6.8 cm (Figure 1A).

**Figure 2:**
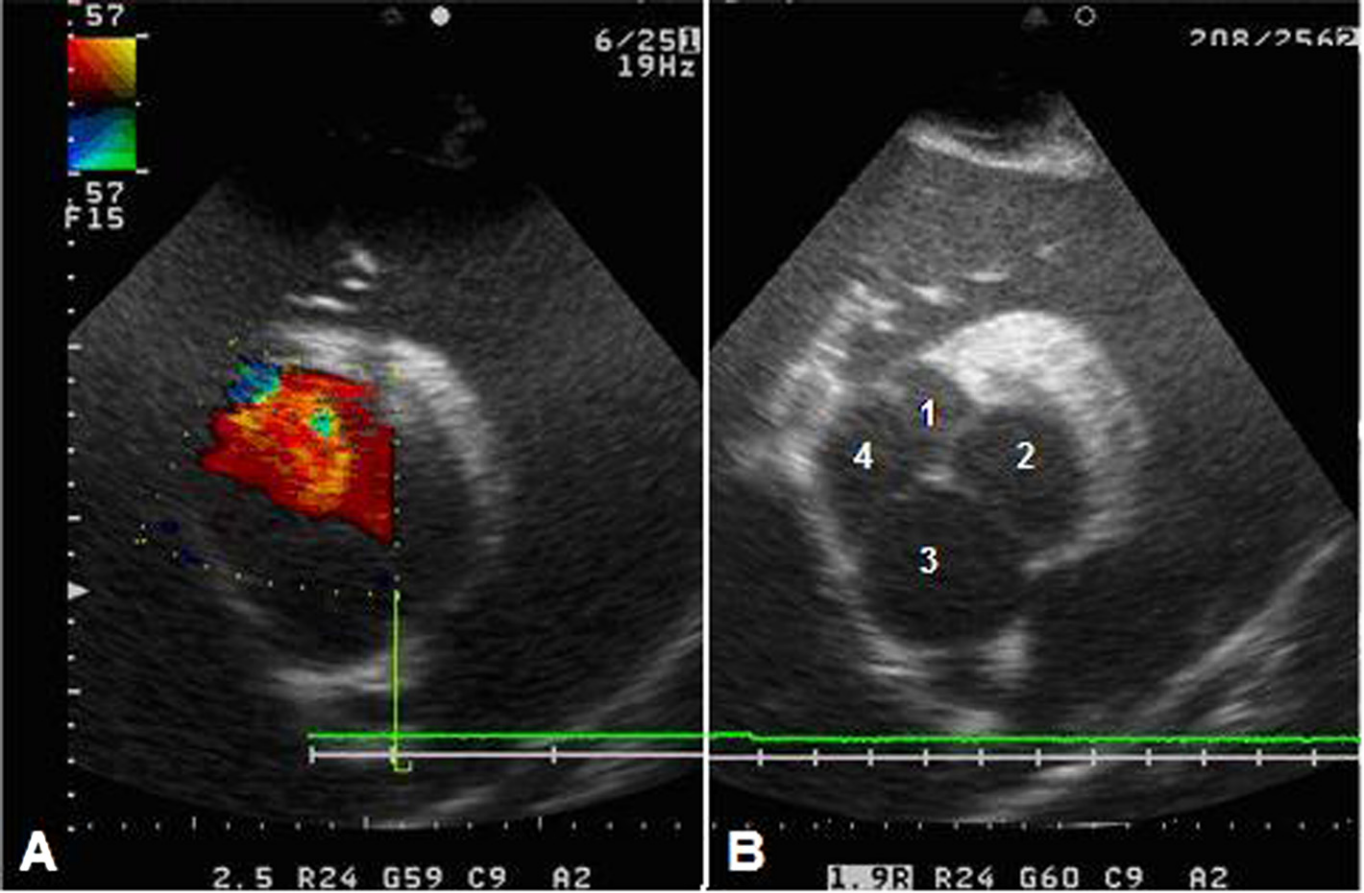
**Quadricuspid aortic valve. A:** A short-axis image of the left ventricular outflow tract which was obtained by transthoracic echocardiography when the transducer was placed in the third right intercostal space. The mild aortic regurgitation can be seen by color Doppler when the transducer was placed in the third right intercostal space. **B:** A short-axis image of the left ventricular outflow tract which was obtained by transthoracic echocardiography when the transducer was placed in the fourth right intercostal space. A quadricuspid aortic valve (QAV) with one small cusp (1), two equal-sized cusps (2, 4), and one large (3) cusp (a class D QAV according to the classification of Hurwitz and Roberts [1] can be seen in the center of the echocardiogram.

Aliasing of the color Doppler image occurred when transthoracic spectral Doppler echocardiography was carried out in the pulsed wave mode, because the rate of left-to-right shunting of blood through the VSD was high. Accordingly, transthoracic spectral Doppler echocardiography was carried out in the continuous-wave mode in order to obtain accurate measurements of the velocity of the jet (Figure 1C). The maximal velocity of the flow through the VSD, measured by continuous wave Doppler, was 6.72 m/s, reflecting normal left- and right-sided pressures (Figure 1C). The observed abnormalities were accompanied by mild aortic valve regurgitation (Figure 2A) and severe tricuspid regurgitation. An estimation of the severity of aortic insufficiency was performed by determining the area of regurgitation using color Doppler. Three jet sizes have been described in literature: a mild- jet extends just beyond the aortic valve and its height is less than 24% of left ventricle outflow tract, the moderate jet of insufficiency extends to mitral valve tips and the jet width ranges from 25-46% of the left ventricle outflow tract, whereas the severe jet extends deep into the left chamber and the jet width exceeds 47% of the left ventricle outflow tract [20].

The tricuspid regurgitation systolic murmur was covered by a loud VSD murmur. An assessment of the severity of the tricuspid valve incufficiency was performed by determining the area of regurgitation using color Doppler compared to the total area of right atrial size: mild - under 20%, moderate - 20-50% and severe over 50% [20].

The evaluation of cardiac structures was performed using B- and M-mode echocardiography. The results of the M-mode measurements are presented in Table 1. The normal echocardiographic measurements are based on the study of the horses weighted 274-469 kg [21]. The images revealed an increase in the left ventricular internal diameter and hypertrophy of the left ventricular free wall. In the short-axis view, four aortic valve cusps:one small cusp, two equal-sized cusps, and one large cusp, were detected (Figure 2B). B-mode views revealed normal morphology of the atrioventricular valves and atrial size.

**Table 1:** **The measured cardiac dimensions and normal echocardiographic M-mode values in horses**[21]

**Variable**	**M-mode results for the patient (430 kg)**	**Normal echocardiographic measurements in horses 274-469 kg**
		**(Mean ± SD)**
Interventricular septal thickness at end-diastole (cm)	2.74	2.4 ± 0.2
Interventricular septal thickness at end-systole (cm)	3.8	3.8 ± 0.5
Left ventricular internal diameter at end-diastole (cm)	12.81	8.9 ± 1.4
Left ventricular internal diameter at end-systole (cm)	9.01	5.9 ± 0.9
Left ventricular free wall thickness at end-diastole (cm)	2.39	2.2 ± 0.5
Left ventricular free wall thickness at end-systole (cm)	4.59	2.7 ± 0.8

Despite the stallion’s relatively poor condition, the progressive loss of body mass, the presence of subcutaneous edema and cardiomegaly with VSD and QAV, and normal results of the laboratory examination, the castration was performed under general anaesthesia. Pre-anesthetic sedation was achieved with romifidine (0.06 mg/kg, Sedivet, Boehringer Ingelheim) followed by butorphanol (0.02 mg/kg, Butomidor, Richter Pharma), and both drugs were administered intravenously slowly over several minutes. Anesthesia was induced with a combination of intravenously-administered diazepam (0.1 mg/kg, Relanium, Polfa S.A.), propofol (0.5 mg/kg, Propofol-Lipuro, B-Brown), and ketamine (1.5 mg/kg, Vetaketam, Vet-Agro). After endotracheal intubation, anesthesia was maintained for about 80 minutes using isoflurane in oxygen and a constant infusion of ketamine (3 mg/kg/hour for the first 40 minutes and then progressively reduced to 0.75 mg/kg/hour, Vetaketam, Vet-Agro), propofol (0.6 mg/kg/hour, Propofol-Lipuro, B-Brown), and fentanyl (8 μg/kg/hour, Fentanyl WZF, Polfa S.A.) with intermittent positive pressure ventilation. Following an intratesticular injection of lidocaine under anesthesia and an intramuscular injection of procaine benzylopenicillin (6.5 mg/kg, Penicillin L.A., ScanVet) and benzathine benzylopenicillin (4.5 mg/kg, Penicillin L.A., ScanVet), a bilateral castration was performed in dorsal recumbency. The anaesthesia and the recovery from anesthesia were uneventful and the wound healed without complications. Post-operative medications included romifidine (0.01 mg/kg, Sedivet, Boehringer Ingelheim) and butorphanol (0.05 mg/kg, Butomidor, Richter Pharma), which were administered while the horse was in the recovery box. Procaine benzylopenicillin (6.5 mg/kg, Penicillin L.A., ScanVet) and benzathine benzylopenicillin (4.5 mg/kg, Penicillin L.A., ScanVet) were administered intramuscularly q 24 hours for three days after surgery, and flunixin meglumine (1.1 mg/kg, Finadyne Solution, MSD Animal Health) was administered intravenously q 24 hours for five days. The patient required further hospital hoof care because of equine canker and was discharged one month after the surgery. The gelding was euthanasied 17 months after surgery because of a progressive loss of body weight, weakness and recumbency.

## Discussion

The cusps or leaflets of a QAV are formed early in embryonic development with the formation of a pair of mesenchymal ridges in the cephalad portion of the truncus arteriosus [2]. The aortic valve is formed after the development of the main bulbar ridges, which then fuse to give the aorticopulmonary septum. In the aortic trunk, three subendothelial swellings appear and grow to form the triangle-shaped valve, and alterations in its division may cause a dissymmetry, which results in four valve cusps in the aortic trunk [7].

Although QAV is considered to be a rare cardiac malformation (0.008-0.033% of the population) [22], numerous case reports of QAV have been published in human medical literature. QAV usually occurs as a single defect, and, less often, in combination with other congenital cardiac defects [22,23]. QAV may also be associated with an anomaly of coronary arteries (10% of cases of QAV) [24], patent ductus arteriosus, VSD, pulmonary stenosis, supravalvar aortic stenosis, subaortic stenosis, hypoplasia of the anterior leaflet of the mitral valve, perforation of the cusps, aneurysms of the Valsalva sinus, left superior vena cava, hypertrophic cardiomyopathy, atrioventricular block or double right kidney [7,22,23,25-29]. In human medical literature, VSD and aortic arch anomalies are shown to be associated with or be a part of congenital syndromes such as Pierre-Robin syndrome, DiGeorge syndrome [30-32] and velo-cardio-facial syndrome [33], which have not been described in horses.

In veterinary literature, reports of the occurrence of QAV are scarce when compared to the number of published reports on QAV in humans. There have been reports of QAV as a single defect and or it co-occurring with VSD or other cardiac defects (an enlarged ostium of the left coronary artery, mitral valve degeneration, and patent ductus arteriosus) in dogs [2-4]. QAV has also been reported in the Syrian hamster [6] and in an adult great white-toothed shrew, *Crocidura russula *[5].

In humans, QAV is diagnosed more often in men than in women [34]. QAV has been found as a single defect exclusively in male dogs [2]. Although this case was a stallion, it is not possible to definitively determine whether a sex predisposition for QAV exists in horses, since a QAV has not been previously reported in horses.

QAVs have a diverse morphology. Hurwitz and Roberts [1] classified QAVs into seven types (from A to G) according to the relative size of the four cusps [1]. According to this classification, a class A QAV has four cusps of similar size, a class B QAV has three cusps of the same size and one small cusp, a class C QAV has two small equal-sized cusps and two large equal-sized cusps, a class D QAV has a large cusp, two medium-sized cusps, and one small cusp, a class E QAV has three equal-sized cusps and one large cusp, a class F QAV has two large equal-sized cusps and two small unequal-sized cusps, and a class G QAV has four cusps, each of which is of unequal size. According to this classification, the care-report has a class D QAV. In humans, the most common types of QAVs are class A, B, D, and G [22,35]. Only QAV class A, B, and C have been described in dogs [2-4].

One of the outcomes of having a QAV with cusps of different sizes is valvular malfunction, which leads to their fibrous thickening and incomplete coaptation of the cusps. Consequently, mild to moderate regurgitation may result [22,23,35,36], but it may not be possible to echocardiographically differentiate between congenital malformation of the leaflets and other causes of aortic insufficiency [20]. It should be noted that progressive aortic regurgitation is usually observed in patients with a class B QAV [22]. Aortic valve function may remain unchanged in individuals with a class A QAV [26]. In this horse, which had a class D QAV, we attributed the mild silent aortic regurgitation that was observed on echocardiography to the presence of an additional and abnormal aortic cusp. This cusp caused an unequal distribution of shear stress and abnormal leaflet coaptation.

The evaluation of the aortic regurgitation may be multifarious. Colour Doppler echocardiography is very useful in showing an area occupied by the regurgitant jet. A spectral Doppler method can be used to assess pressure gradients between the aorta and left ventricle. In human medicine, maximal velocity and pressure half-time of the regurgitant blood flow can be related to the severity of its consequences such as ventricular volume overload. Unfortunately, critical prospective studies have not validated the reliability of these variables in the horse [37]. Aortic regurgitation in the presented horse was mild. The regurgitant jet was very small and it was not easy to obtain an adequate image with a spectral Doppler. Due to the fact that it would most likely be encumbered with an error, we decided to forfeit this measurement.

A QAV is usually diagnosed on echocardiography following the hearing of a heart murmur and, in most documented cases of QAV in humans, the patients are asymptomatic before the diagnosis of QAV [7]. Occasionally, patients complain of difficulty in breathing, palpitations, and episodes of syncope or pain below the sternum [26]. The owner of the horse found no exercise intolerance before castration, probably because the patient was kept as a pet. It should be noted that no exercise tests were performed in the horse after its admission and it was not subjected to any form of training prior to surgery. Serres reported that dogs with an aortic valve defect and a patent ductus arteriosus displayed clinical signs, such as exertional intolerance and growth retardation [4].

VSDs have been described in both human and veterinary medical literature and their formation is associated with an abnormal embryonic conjunction of membranous and muscular portions of the intraventricular septum [38]. In physiological conditions, the large muscular portion of the interventricular septum is formed by the condensation of the trabeculae [38]. On the other hand, the endocardial cushion, created by mesenchymal cells derived from endocardial cells, expands to fuse with the muscular septal part and aorticopulmonary septum, forming a membranous portion of the interventricular septum [38].

In human as well as in veterinary literature, VSDs are classified according to their location. They can be muscular when the VSD is located in the trabecular part of the outflow or outlet septum or membranous when the VSD is located directly below the aortic or tricuspid valve or is connected to both valves. According to Reef [39], VSDs in horses, dogs, and cats are usually located in the membranous septum of the outflow tract of the left ventricle, just below the right coronary cusp of the aortic or tricuspid valve. In horses, the most commonly diagnosed congenital heart disease is a VSD. It may occur as a single defect, although it is often encountered in certain congenital syndromes, such as the tetralogy of Fallot [11,15-17]. Arabian horses are predisposed to VSD [9]. Marr [37] reported that Welsh Mountain ponies were more predisposed than Thoroughbreds. Most VSDs are located in the membranous portion of the intraventricular septum, directly below the right coronary cusp of the aortic valve, and are often the cause of aortic valve regurgitation [37]. Although this defect has also been described to occur in the intraventricular septum directly below the tricuspid valve, the frequency of its occurrence at this location is less than that of VSDs that are found directly below the right coronary cusp of the aortic valve [37]. Rare cases of a muscular VSD and VSDs which are directly connected to the aortic valve cusps have been reported in horses [40]. When such VSDs are present, moderate to severe regurgitation or even aortic valve cusp rupture may develop. The presence of a VSD usually results in a left-to-right jet, which was present in the described horse. However, the flow may be bi-directional in the presence of another congenital cardiac abnormality, such as a bicuspid pulmonary valve [37]. VSD may also result in a significantly increased flow through pulmonary vasculature, especially in the case of high sided VSD. This leads to pulmonary hypertension called Eisenmenger’s Syndrome, but this phenomenon was not detected in the gelding.

Eccentric enlargement, left ventricle dilatation and normal wall thickness to chamber size ratios occur in patients with VSD, suggesting adequate compensatory hypertrophy [41]. These findings were detected in the described horse. Probably it was not closely assosiated with aortic regurgitation because of the mild type of the valvular insufficiency and no presence of early closure of the mitral valve (before QRS complex). The significant increase in the size of the ventricle can also cause a dilation of the aortic root [42]. However, the aortic diameter in the gelding was normal.

Although animals with VSD may be asymptomatic, the presence of a VSD can be the cause of exercise intolerance and left ventricular heart failure [37]. As already noted, the owner reported that the horse had not been trained. The only clinical signs of cardiac failure that were found in the presurgical clinical examination were subcutaneous edema and poor growth. The muscle loss was attributed to hypertrophic pododermatitis, which caused problems with movement and food intake, although cardiac failure cannot be ruled out as a possible cause. The presence of the QAV and VSD was detected using echocardiography, which is currently considered to be the gold standard non-invasive method for the reliable diagnosis of cardiac malformations and functional anomalies. It is specifically useful in detecting the anatomy and the presence of any defects of the aortic valve, which can be best visualized when the images are obtained in the right parasternal short-axis view using two-dimensional echocardiography. The presence of cardiac shunts, such as a VSD, can be confirmed by transthoracic color Doppler and spectral Doppler echocardiography, and are best visualized in the parasternal long four-chamber view and short-axis view of the right outflow tract. Accordingly, invasive cardiac catheterization and cardiac ventriculography are no longer needed to detect cardiac malformations and functional anomalies [43].

A crescendo–decrescendo systolic murmur (holosystolic murmurs) due to VSD, which is detected during auscultation, is best heard in horses on the right side of the chest. It is also audible on the left side in the third intercostal space. The VSD type of murmur is an ejection murmur. It is a sound that is generated due to the two abnormal turbulent flows: (a) from the left to the right ventricle through the VSD and (b) functional stenosis of the pulmonary artery caused by an increased volume of blood. The murmur in the described horse was caused by a shunt in the interventricular septum and was best heard on the right side of the chest at the base of the heart.

Some congenital defects may have a genetic origin. Prior to the cardiac examination and castration, the stallion had successfully impregnated two mares, and we monitored these two pregnancies. One of the pasture-kept mares miscarried in the late stage of the pregnancy, and the aborted fetus was not found. The second mare bore a healthy foal with no physical or cardiac abnormalities. Until cardiac examinations are conducted on the horse’s ancestors and relatives, the possibility that a genetic origin underpins the horse’s cardiac abnormalities cannot be ruled out.

The prognosis for VSD depends on the size of the VSD, its location, the maximum flow rate through the VSD and the exercise requirements of the horse. According to long-term studies of horses with congenital heart disease, horses with a VSD usually survive for at least eight years [9]. A prognosis in the small breeds of horses and ponies with a VSD is usually based on its size. However, comparing the size of VSD to the size or diameter of the aortic root can allow additional information about the long-term outcome in horses with this disorder. Using this latter method, the prognosis for horses with a small VSD in the outflow tract of the left ventricle and without heart failure and exercise intolerance is usually good, especially when the diameter of the VSD is less than one-third of the diameter of the aortic root [36,37,44]. Reef [40] conducted a study on standard size horses, predominantly Thoroughbreds and Arabian horses, and found that all horses with a VSD in the upper part of the septum, whose maximum diameter was smaller than 2.5 cm, and whose maximum flow rate through the VSD was greater than 4 m/s, had a successful sport career. Using these measurements, the prognosis for the presented horse could be considered relatively good, since the VSD/aorta root ratio was 0.24, the diameter of the VSD was less than one-third of the aortic root diameter in diastole and the shunt velocity was greater than 4 m/s (6,72 m/s). However, our prognosis for the patient was cautious because of an additional presence of valvular regurgitations, cardiomegaly, progressive loss of muscle mass and equine canker in all four limbs.

In human medicine, valvuloplasty is done in order to correct QAV and its accompanying regurgitation [22,28,45,46], as well as any coexisting heart defects. A VSD in humans can, for example, be percutaneously closed using a coil [7]. We are not aware of any successful attempts to percutaneously close a VSD in a horse using the procedures and materials that have been used in human medicine. However, it has been reported that cardiopulmonary bypass surgery has been used to correct a cardiac defect in a horse [47]. Spontaneous closure of a VSD can occasionally occur, and such closures have been reported in children, foals, dogs, and cats [11,48]. Although the horse’s owner was informed of these surgical treatment options, the owner did not give consent to perform surgery to correct the VSD. Furthermore it is unclear whether a surgical approach would have been useful at this stage of the disease.

## Conclusion

The finding of a QAV in combination with a VSD in a horse is interesting because such a finding, to the best of our knowledge, has not been previously described in the equine literature.

## Abbreviations

VSD: Ventricular Septal Defect; QAV: Quadricuspid Aortic Valve.

## Competing interests

None of the authors has any financial or personal relationships that could inappropriately influence or bias the content of the paper.

## Authors’ contributions

JMN and UP were responsible for the study design. KMM carried out the echocardiographic examination. UP performed the electrocardiographic examination and was responsible for laboratorial parameters. AKB and RZH carried out the surgical castration. MAS was responsible for anaesthesia. KMM and UP drafted the manuscript. JMN, RZH, MAS and AKB were involved in work supervision and writing of the manuscript. All authors read and approved the final manuscript. JMN and UP are guarantors of the paper.
